# Fast and Cost-Effective Genetic Mapping in Apple Using Next-Generation Sequencing

**DOI:** 10.1534/g3.114.011023

**Published:** 2014-07-16

**Authors:** Kyle M. Gardner, Patrick Brown, Thomas F. Cooke, Scott Cann, Fabrizio Costa, Carlos Bustamante, Riccardo Velasco, Michela Troggio, Sean Myles

**Affiliations:** * Department of Plant and Animal Sciences, Faculty of Agriculture, Dalhousie University, Nova Scotia, Canada; † Department of Crop Sciences, University of Illinois, Urbana, Illinois; ‡ Department of Genetics, Stanford School of Medicine, Stanford University, Stanford, California; § Genetics and Molecular Biology Department, IASMA Research Center, San Michele all’Adige, Italy

**Keywords:** next-generation DNA sequencing, genotyping-by-sequencing, apple, *Malus*, QTL, SNP

## Abstract

Next-generation DNA sequencing (NGS) produces vast amounts of DNA sequence data, but it is not specifically designed to generate data suitable for genetic mapping. Recently developed DNA library preparation methods for NGS have helped solve this problem, however, by combining the use of reduced representation libraries with DNA sample barcoding to generate genome-wide genotype data from a common set of genetic markers across a large number of samples. Here we use such a method, called genotyping-by-sequencing (GBS), to produce a data set for genetic mapping in an F1 population of apples (*Malus* × *domestica*) segregating for skin color. We show that GBS produces a relatively large, but extremely sparse, genotype matrix: over 270,000 SNPs were discovered but most SNPs have too much missing data across samples to be useful for genetic mapping. After filtering for genotype quality and missing data, only 6% of the 85 million DNA sequence reads contributed to useful genotype calls. Despite this limitation, using existing software and a set of simple heuristics, we generated a final genotype matrix containing 3967 SNPs from 89 DNA samples from a single lane of Illumina HiSeq and used it to create a saturated genetic linkage map and to identify a known QTL underlying apple skin color. We therefore demonstrate that GBS is a cost-effective method for generating genome-wide SNP data suitable for genetic mapping in a highly diverse and heterozygous agricultural species. We anticipate future improvements to the GBS analysis pipeline presented here that will enhance the utility of next-generation DNA sequence data for the purposes of genetic mapping across diverse species.

The introduction of new high-throughput DNA sequencing technologies has dramatically reduced sequencing costs and increased the pace of genomics research. One of the primary goals of genomics research is to establish relationships between genotypes and phenotypes. In agriculture, such genotype–phenotype associations form the basis of genomics-assisted breeding programs that aim to accelerate the breeding of improved varieties. Long-lived woody perennials are expensive to breed using traditional methods and therefore stand to benefit more from the genomics revolution than most other agricultural species. Offspring from breeding programs can be genetically screened and discarded at the seedling stage without incurring the enormous expense of growing them for years to fruit-bearing maturity for evaluation (Bus *et al.* 2000; Kellerhals *et al.* 2000; Dirlewanger *et al.* 2004; di Gaspero and Cattonaro 2010).

Most forms of genetic mapping require the collection of genome-wide polymorphism data, where genotypes from a set of common loci are obtained from a set of samples. Although next-generation DNA sequencing technologies produce vast amounts of DNA sequence data, they were not designed to generate these types of genotype data. Recently, however, next-generation sequencing technologies have been coupled with reduced representation libraries and DNA barcoding to simultaneously identify and genotype a common set of polymorphic loci across a set of samples in a single experiment. The two most common of these methods include genotyping-by-sequencing (GBS) (Elshire *et al.* 2011) and RADseq (Baird *et al.* 2008), and the present study focuses on GBS. In addition to the low per-sample cost, there are several benefits to using sequence-based genotyping methods over microarray-based technologies (Myles 2013). For example, polymorphism discovery and genotyping are completed in a single step, which not only saves time but also reduces the ascertainment bias inherent in the process of developing genotyping microarrays. Moreover, as reference genomes, alignment methods, and genotype calling algorithms improve, raw sequence data collected today will become more valuable in the future because improved methods will enable more information to be extracted from the original raw files.

Despite difficulties in experimental design, due to self incompatibility and high heterozygosity, there is a wide variety of apple genetic maps constructed from bi-parental crosses. Most of these linkage maps have been built with low-throughput genetic markers such as microsatellites (Celton *et al.* 2009; Fernández-Fernández *et al.* 2012) and AFLPs (Liebhard *et al.* 2003; Kenis and Keulemans 2005), resulting in relatively low marker density across assembled linkage groups. Recently, there has been a shift toward the high throughput identification of single nucleotide polymorphisms (SNPs) in apple spurred on by decreasing DNA sequencing costs and the availability of an apple (*Malus* × *domestica*, cultivar "Golden Delicious") reference genome (Velasco *et al.* 2010). Chagne *et al.* (2012a) detail the creation of a SNP genotyping microarray that assays 8000 SNPs discovered from low coverage sequencing of 27 cultivars. To date, the apple 8K SNP array has been used to create saturated linkage maps in bi-parental cross populations (Antanaviciute *et al.* 2012) and to perform genomic selection (Kumar *et al.* 2012) and genome-wide association (Kumar *et al.* 2013) in diverse breeding material. Although SNP arrays are widely used, the high levels of polymorphism in many agricultural species like apples often result in unreliable or useless genotype calls because of highly variable probe–sequence hybridization ( Miller *et al.* 2013). In addition, the ascertainment bias inherent in the design of SNP genotyping microarrays results in only a small fraction of the queried loci being polymorphic in any given bi-parental cross (Micheletti *et al.* 2011). For example, only approximately one-third of the SNPs on the apple 8K SNP array were observed to be polymorphic in a "Royal Gala" × "Granny Smith" segregating population (Chagne *et al.* 2012a).

It is evident that GBS offers several advantages over competing technologies and is quickly becoming the genotyping method of choice in many agricultural systems (Poland and Rife 2012; Myles 2013). For example, GBS has been recently used for a variety of applications including saturating an existing genetic map in rice (Spindel *et al.* 2013), creating high-density genetic maps in wheat and barley (Poland *et al.* 2012b), performing genomic selection in wheat (Poland *et al.* 2012a), ordering of a draft genome sequence in barley (Consortium 2012; Mascher *et al.* 2013a), and characterizing germplasm diversity in maize and switchgrass (Lu *et al.* 2013; Romay *et al.* 2013). Almost all GBS studies to date have focused on inbred lines, because genotype calling in highly heterozygous crops using next-generation DNA sequence data requires more data and is far more complicated. The present study addresses this issue by presenting a pipeline for GBS SNP calling in apples and follows recently published work on GBS workflows developed for other heterozygous crops like grape (Barba *et al.* 2014) and raspberry (Ward *et al.* 2013). Using a single lane of Illumina HiSeq data, we identified a robust set of SNPs and used them to generate a saturated genetic linkage map of the apple genome and map a major QTL for apple skin color in an F1 population.

## Materials and Methods

### Population description and phenotyping

The "Golden Delicious × Scarlet Spur" population investigated here is planted at the experimental orchard of the Foundation Edmund Mach (FEM) in San Michele all’Adige, Italy. Each individual progeny is represented by a single tree grafted on M9 rootstock and planted in 2003. The population has been grown and maintained following standard agronomical practice for fruit thinning, canopy pruning, chemical fertilization, and disease control. Due to the large variation in ripening time among progeny, phenotyping was repeated three times during the harvesting season. Skin color was scored as a binary trait: trees had apples with either yellow/green skin (like Golden Delicious) or red skin (like Scarlet Spur).

### GBS library construction

GBS libraries were constructed using the two-enzyme modification of the original GBS protocol (Elshire *et al.* 2011; Poland *et al.* 2012b). DNA was extracted using commercial extraction kits. Restriction/ligation reactions were performed in 96-well plates using 500 ng of DNA from each individual, digestion with *Hin*dIII-HF and *Msp*I (New England Biolabs, Ipswich, MA), and 0.1 μM and 10 μM of A1 and A2 adapters per well, respectively. Libraries were pooled, size-selected on a 1% agarose gel, column-cleaned using a PCR purification kit (Qiagen, Valencia, CA), and amplified for 12 cycles using Phusion DNA polymerase (NEB). Average fragment size was estimated on a Bioanalyzer 2100 (Agilent, Santa Clara, CA) using a DNA1000 chip following a second column-cleaning, and library quantification was performed using PicoGreen (Invitrogen, Carlsbad, CA). Pooled libraries were adjusted to 10 nmol and sequenced with 100-bp, single-end reads on the HiSeq2000 (Illumina, San Diego, CA).

### SNP calling

We created a custom bioinformatics pipeline using custom Python scripts and existing software to process raw GBS sequence data from a single lane of an Illumina HiSeq sequencer into SNP genotype tables (Supporting Information, Figure S1). DNA barcode deconvolution and basic sequence quality filtering was performed using a custom Python program (barcode_splitter.py). This program splits the raw Illumina fastq file into 96 separate fastq files based on the barcode sequences associated with each sample while filtering out reads containing any ambiguous bases in the barcodes or restriction site remnants immediately after the barcode sequence. All sequences successfully passing these basic filters were then scanned for the presence of an additional restriction site remnant and, if present, the read was trimmed accordingly. Reads were also trimmed if the sequence contained the common (A2) GBS adapter, indicating that the genomic fragment sequenced was less than 90 to 100 bp in size.

The separate fastq files for each DNA sample were then aligned to version 1.0 of the apple reference genome (Velasco *et al.* 2010) using bwa (Li and Durbin 2009), allowing a maximum of 4% sequence mismatch. Alignments were converted to the SAM format, then merged and sorted into one master binary alignment file (BAM format) with SAMtools 0.1.18. (Li *et al.* 2009). SNP calling was performed using the genome analysis toolkit (GATK; McKenna *et al.* 2010) on the BAM file using a minimal set of filters that required a called SNP to have a locus quality score of at least 30 given a prior probability of heterozygosity of 0.01.

### SNP filtering and segregation analysis

SNP calls were filtered for quality by restricting the marker set to biallelic SNPs, requiring genotype calls at each SNP to have a depth of coverage of six reads in each sample, implementing a minor allele frequency threshold of ≥0.2 and limiting missing genotype data to a maximum of 20% per SNP. All filtering was performed using vcftools 0.1.10 (Danecek *et al.* 2011) and the final SNP genotype tables were output into PLINK format (Purcell *et al.* 2007). The final SNP table contained 3967 SNPs and all of these SNPs were used to map apple color using a simple chi-squared test (see below). The construction of the genetic map, however, required further filtering based on the segregation pattern of the genotypes in parents and offspring.

Linkage mapping in apple F1 populations is referred to as a double pseudo-testcross and only three genotype combinations in the parental lines are informative for the construction of a genetic map: when one parent is heterozygous and the other is homozygous (*i.e.*, either AA × AB or AB × AA) and when both parents are heterozygous (*i.e.*, AB × AB). Only SNPs following these segregation patterns in the parents were retained. Each SNP was subsequently tested for the expected segregation ratios in the F1 progeny: heterozygous in Golden Delicious (1:1); heterozygous in Scarlet Spur (1:1); and heterozygous in both Golden Delicious and Scarlet Spur (1:2:1). SNPs deviating from these ratios according to a chi-squared test (*P* < 0.01) were not included in map construction. Finally, progeny genotypes inconsistent with Mendelian inheritance from the parental genotypes were set to missing. After implementing these filters, 2436 SNPs remained for linkage map construction.

### Linkage map construction

A single composite linkage map was constructed using JoinMap 4.0 (Stam 1993) by combining the backcross type (Aa × aa) markers segregating within each parental background and intercross type (Aa × Aa) markers segregating within both parental backgrounds. Markers suspected of being incorrectly phased by JoinMap, due to high pairwise linkage LOD and spurious recombination fractions above 0.5, had their allele codes switched manually but were dropped from further analysis if phasing problems persisted. To increase mapping efficiency, pairs or groups of loci with identical genotypes (*i.e.*, complete linkage) were identified and a single marker was chosen to represent the group.

Linkage groups were constructed and ordered with a linkage LOD of at least 6.0, a minimum recombination fraction of 0.35, and a jump threshold of 5. SNPs exhibiting “suspect linkage” to several loci within their assigned group (as determined by JoinMap), poorly fitting markers within an ordered group, and markers that greatly inflated the linkage group size were dropped from the final mapping. All map distances were calculated using the Kosambi mapping function. After applying these filters, 1994 SNPs remained within the genetic linkage map.

### Mapping skin color

For association analysis based on the physical coordinates obtained from the reference genome, the full set of 3967 SNPs were tested for association with skin color measurements using the case/control single marker analysis in PLINK 1.04 (Purcell *et al.* 2007), which uses a chi-squared test of independence of allele frequencies between cases and controls with one degree of freedom at each marker independently. For linkage map–based QTL analysis, R/qtl 1.26-14 (Broman *et al.* 2003) was used with the single binary QTL interval mapping model, scanning the linkage map at 1-cM steps for the presence of a significant QTL. A significance threshold of 3.05 was determined by permutation tests on 1000 randomizations of the trait data (Churchill and Doerge 1994). The PLINK genotype files, JoinMap input files, and phenotype data are available from the Dryad Digital Repository (http://doi.org/10.5061/dryad.55t54).

## Results

### Alignment of GBS reads to the reference genome

GBS of a single plate of 96 DNA samples yielded 85,129,960 100-bp reads using Illumina HiSeq 2000 sequencing technology. The high proportion of reads beginning with a barcode sequence (98.2%) and containing a restriction site remnant (99%) indicated that the library preparation was effective and the data were of high quality. In addition, there were very low occurrences of chimeric reads (∼1.0%) and of reads containing downstream adapter sequences (∼1.2%).

**Figure 1:**
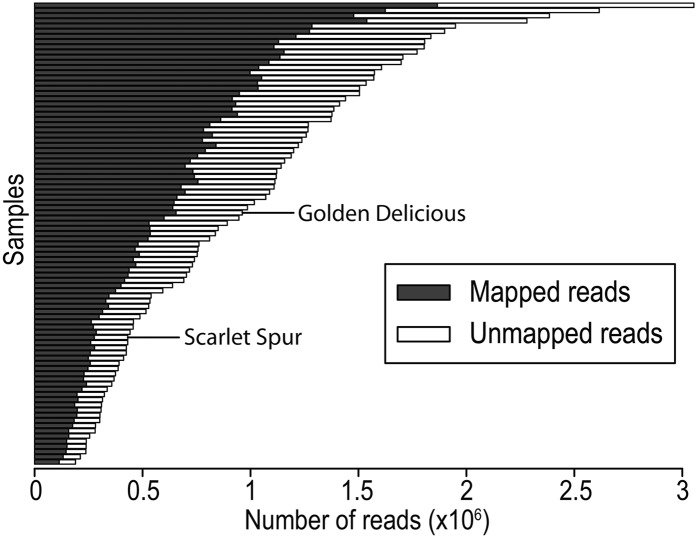
Results of alignment of GBS reads to the apple reference genome. For each sample, the number of reads mapped and number of reads unmapped to the reference genome are shown. The read counts for the parents of the F1 mapping population, Golden Delicious and Scarlet Spur, are indicated.

After aligning each of the 96 samples’ reads to the "Golden Delicious" v1.0 reference genome, seven of the samples were found to have a relatively low numbers of reads (<150,000) that uniquely aligned to the genome and these samples were subsequently dropped from further analysis. In the remaining 89 samples (87 F1 progeny and 2 parents) there was an average of 973,896 (SD = 609,869) reads per sample and an average of 628,085 reads (SD = 393,153) uniquely aligned to the reference genome (Figure 1). Despite the wide range of read counts across samples, there was relative uniformity in the proportion of reads successfully mapped across samples with an average of 63.5% (SD = 2.5%).

### SNP calling

**Figure 2:**
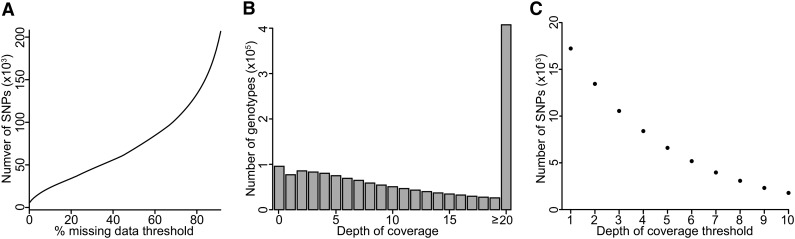
SNP and genotype counts from GBS data. (A) Cumulative count of SNPs identified across varying missing data thresholds. More than 200,000 SNPs are called with a very liberal missing data threshold of 90%, but only 30,393 SNPs remain if only SNPs with <20% missing data are retained. (B) The number of genotypes called at increasing levels of sequencing depth, after retaining only SNPs with <20% missing data. (C) The number of SNPs retained at increasing minimum thresholds of sequence depth while retaining only SNPs with <20% missing data. Here, we chose a minimum depth of coverage of six reads. Thus, only SNPs with at least six supporting reads and <20% missing genotypes were retained, resulting in a set of 3967 SNPs.

Considering only the successfully mapped reads from 89 samples, SNPs were discovered and genotypes were called by analyzing the single master alignment file with GATK (McKenna *et al.* 2010). After using a minimal set of initial quality filters (see *Materials and Methods*), 273,835 SNPs were identified. However, the resulting genotype matrix was extremely sparse: more than 75% of the 273,835 SNPs contained >50% missing genotypes (Figure 2A). Restricting the analysis to SNPs with <20% missing genotype data drastically reduced the number of SNPs to 30,393. It is likely that many false-negative genotype calls still exist in the resulting genotype table because confidently calling heterozygotes in a highly heterozygous diploid species like apple requires a relatively high depth of sequence coverage compared with genotype calling in inbreds. For the set of 30,393 SNPs, the number of genotype calls at various sequence depth thresholds is shown in Figure 2B. Despite the observation that many genotype calls are supported by >20 reads, the number of SNPs for downstream analysis declines rapidly as the minimum depth of coverage threshold is increased from 1 to 10 while implementing a missing data threshold of 20% (Figure 2C). We further reduced the number of markers to 3967 SNPs by applying a minimum depth of coverage threshold of six sequence reads for a genotype call (*i.e.*, any genotype with fewer than six supporting sequence reads was set to missing) and then re-filtering for SNPs with <20% missing genotypes. The choice of six reads per genotype as a threshold was arbitrary: it was chosen as a tradeoff between increased confidence in genotype calls and the number of SNPs retained for mapping. This set of 3967 SNPs was used to map skin color using a chi-squared test. To create the genetic map, a final set of 2436 SNPs was retained after filtering for Mendelian inconsistencies and segregation distortion (see *Materials and Methods*).

### Genetic map construction

The final set of 2436 SNPs was separated into the three segregation types: 884 heterozygous in Golden Delicious, 1044 heterozygous in Scarlet Spur, and 508 heterozygous in both parental varieties. Once this set of SNPs was imported into JoinMap 4.0, 442 segregated identically to at least one other SNP (*i.e.*, no recombinants observed) and were dropped from further mapping. The remaining 1994 SNPs were successfully grouped into 17 linkage groups (Figure S2), as expected for the apple genome, at a conservative LOD threshold of six. Only 35 loci were found to be unlinked to any group, suggesting a high degree of saturation of the assembled mapping groups. Once ordered, the linkage groups spanned 1272 cM, with individual linkage groups ranging from 56 cM to 96 cM. Across linkage groups, the average marker density was high with a marker found every 0.68 cM (± 0.13 SD).

### QTL mapping for apple skin color

**Figure 3:**
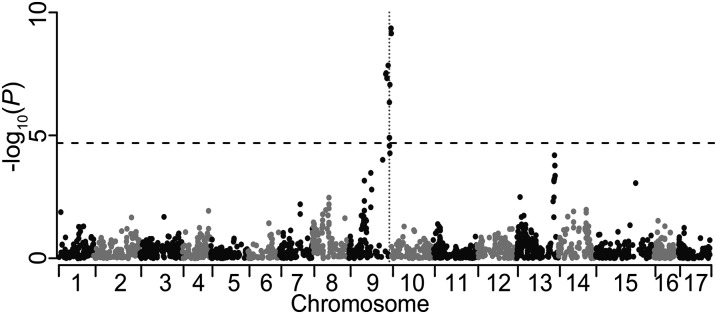
Manhattan plot of a single marker association analysis for apple skin color. Each of the 3967 SNPs is plotted according to its physical position from the "Golden Delicious" reference genome and the −log_10_ *P* value of the single marker association test. The horizontal dotted line represents the Bonferonni-corrected *P* value significance threshold. The vertical dotted line represents the location of the MYB transcription factor gene known to be responsible for skin color variation.

Single marker analysis using the final set of 3967 SNPs identified 15 SNPs significantly associated with skin color after applying a conservative Bonferroni correction for multiple testing (*P* < 1.26 × 10^−5^) (Figure 3). These SNPs cluster within a single interval on chromosome 9 (bounded by coordinates 29,305,493 and 33,701,563). There was also a cluster of SNPs on the distal portion of chromosome 13 that showed a suggestive association with skin color, but all fell short of the corrected significance threshold (Figure 3).

**Figure 4:**
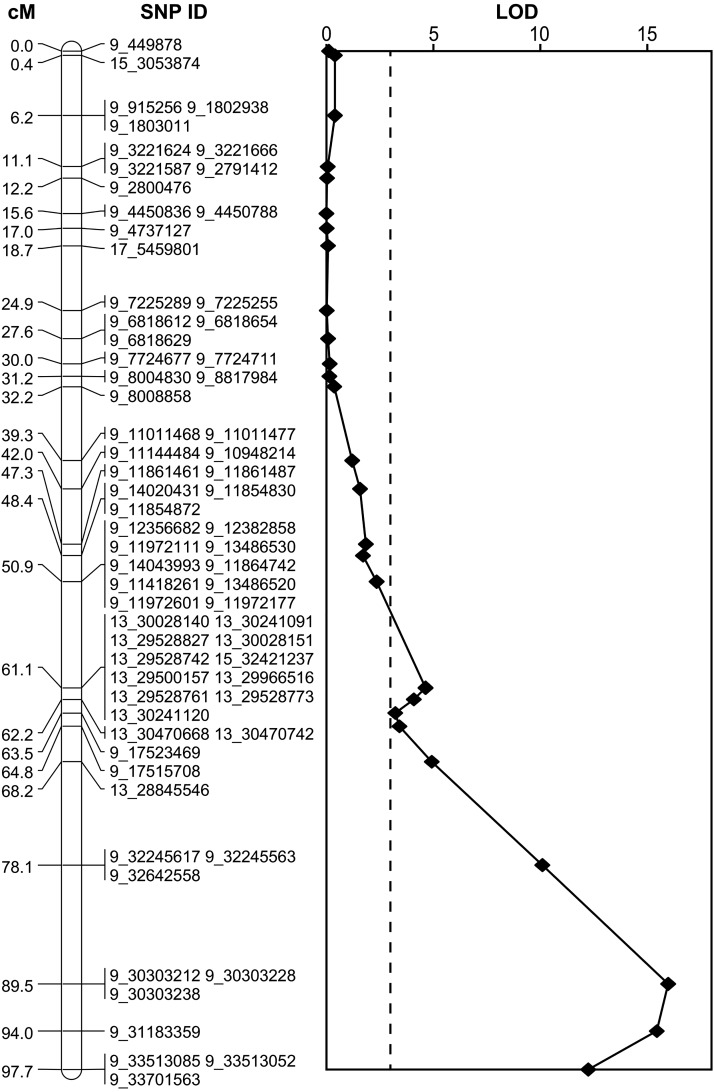
Result of QTL analysis across the linkage group corresponding to chromosome 9 of the apple genome. The left panel indicates the genetic map positions in cM of each of the SNPs or groups of SNPs. Each SNP’s ID indicates its physical position according to the reference genome, *i.e.*, the physical coordinate it was assigned through alignment and SNP calling (*e.g.*, SNP 9_ 449878 mapped to position 449878 on chromosome 9 of the "Golden Delicious" v1.0 reference genome). Note that many SNPs genetically mapping to the linkage group corresponding to chromosome 9 are assigned to other chromosomes according to the reference genome (*e.g.*, SNP 13_30028140). The right panel displays the LOD scores from a QTL analysis for skin color for markers that segregate in the Scarlet Spur genetic background. The horizontal dashed line represents the significance threshold determined by permutation. LOD scores across all linkage groups are shown in Figure S3.

Interval mapping using 1994 SNPs detected one highly significant QTL for skin color on the distal portion of chromosome 9, at position 89.5 cM, near SNP 9_30303238 (Figure 4). The “2 LOD” confidence interval for this QTL extended to a 14-cM region around the point estimate of the QTL position.

## Discussion

A major consideration when conducting GBS experiments is the choice of restriction enzyme used to generate the reduced representation library (RRL). This choice determines the tradeoff between the number of fragments in the library and the sequencing depth of the fragments. There are several GBS library preparation methods currently in use, including the original single enzyme *Ape*KI protocol described by Elshire *et al.* (2011), a double digest using enzymes with differing restriction site lengths (Pst I/Msp I) (Poland *et al.* 2012b) and a multi-step procedure that combines the double restriction enzyme digest with selective PCR amplification (Sonah *et al.* 2013). Although there was ultimately no explicit test of library fragment composition in the present study, using the *Hin*dIII-*Msp*I enzyme combination followed by size selection we observed a very small proportion of sequence reads that required adapter trimming (1.2%), suggesting that most fragments were larger than 100 bp and were thus suitable for GBS. However, the number of DNA sequence reads across individual samples varied by more than an order of magnitude (0.15 million–3 million) (Figure 1), and we ultimately excluded the sequence data of seven samples from analyses due to low read counts. These observations highlight the sensitivity of GBS to DNA sample uniformity. Recent GBS library preparation methods have been shown to improve the uniformity in read counts across samples (Sonah *et al.* 2013), and further improvements are expected.

Because of the apple’s high heterozygosity and ancestral polyploidy, there are major challenges in the assembly of its genome. It is estimated that the assembled portion of the current apple reference assembly represents only 71% of its ∼750-Mb genome (Velasco *et al.* 2010). Despite this constraint, we restricted our analysis to sequences aligning to this assembly to enable a comparison of our inferred linkage groups to the physical map. Approximately 40% of the sequence reads did not map to the assembled genome and were thus excluded from further analysis (Figure 1). These excluded reads are a combination of DNA sequences from ∼29% of the genome that is not represented in the current assembly, DNA sequences that mapped to multiple locations, and sequences that map to a unique position but with too many mismatches (>4 mismatches per 100 bp). As improvements are made to the genome assembly and to DNA sequence length and quality, we anticipate significant improvements in the mapping step of the GBS analysis pipeline. It is worth noting that a potential alternative is to avoid the use of a reference genome altogether and to use a SNP calling pipeline that does not rely on a reference genome (Lu *et al.* 2013).

There is a larger number of possible paths one can take from a DNA sequence file to a SNP genotype table, and it is known that alignment and genotype calling parameters have a strong effect on the resulting quantity and quality of genome-wide SNP data (Mascher *et al.* 2013b). Although established software does exist for SNP genotype calling from GBS data (Bradbury *et al.* 2007), our goal here was to demonstrate that, with simple heuristics applied together with standard software packages, one can generate SNPs of sufficient quality and quantity to be of utility for genetic mapping. Regardless of what tools are used, it is evident that GBS generates a sparse genotype matrix due to uneven sequence coverage across samples and sites: many SNPs are discovered, but genotypes for these SNPs are most often generated from only a small proportion of the DNA samples. For example, more than 250,000 SNPs were discovered in the present study, but when SNPs with >20% missing data are excluded, the number of SNPs remaining for analysis is reduced to ∼30,000 (Figure 2A). Moreover, the number of SNPs decreases as the depth of coverage filter is increased (Figure 2, B and C). We chose arbitrarily to include only genotypes with six or more supporting reads as a trade-off between genotype quality and quantity. Using these thresholds, we obtain a set of 3967 SNPs derived from only 6% of the sequence reads. Thus, in the end, 94% of the sequence we collected in this experiment was ignored. Most GBS studies to date have focused on genotyping inbred lines, which requires far less data and is statistically far simpler than genotype calling in highly heterozygous species like apple, or in polyploids, which are common among species of agricultural importance. GBS SNP calling pipelines designed specifically for highly heterozygous and polyploid species that take haplotype phasing and imputation into account promise to significantly enhance the utility of GBS (Lu *et al.* 2013; Ward *et al.* 2013).

Despite the high proportion of sequence reads discarded due to filtering, there remained a sufficient number of markers to perform genetic mapping of apple skin color with a modest sample size. By mapping skin color, we have intentionally focused on a trait with a simple genetic architecture, which deviates from most QTL studies that focus on more complex traits. Skin color is known to be controlled almost entirely by a single locus of large effect (*i.e.*, effectively Mendelian), and we leverage this knowledge here to verify the utility and power of GBS to identify this known locus. With 3967 SNPs genotyped in 89 samples, a simple chi-squared test for association revealed a single significant peak from position 29.3 Mb to 33.7 Mb on chromosome 9 (Figure 3). This peak overlaps with the R2R3 MYB transcription factor gene at position 32.8 Mb on chromosome 9 known to regulate apple skin color (Takos *et al.* 2006; Espley *et al.* 2007; Lin-Wang *et al.* 2011; Zhu *et al.* 2011; Chagne *et al.* 2013). In addition, a set of 1994 SNPs was used to produce a well-saturated linkage map spanning 1272 cM (Figure S2). This map size is consistent with the sizes of apple genetic maps from previous studies, for example, 1538 cM (Khan *et al.* 2012), 1005 cM (Chagne *et al.* 2012b), and 1143 cM (Han *et al.* 2011). On average, the genetic map has a marker every 0.68 cM, which is in line with the marker density achieved using the apple 8K SNP array (0.5 cM/marker in Antanaviciute *et al.* 2012; 0.88 cM/marker in Chagne *et al.* 2012a). Using this genetic map, interval mapping revealed one highly significant QTL for skin color centered at position 89.5 cM (Figure 4; Figure S3), in agreement with the results from the chi-squared test for association. Thus, with a set of simple heuristics for calling genotypes from GBS data, a saturated genetic map can be generated and QTL mapping can robustly identify genotype–phenotype associations.

In the present study, the parents of the F1 population were included only once and had average sequence coverage (Figure 1). However, because inclusion of a SNP in the genetic map relied on accurate genotype calls from both parents, it may be advisable to sequence the parents of mapping populations to a higher depth, *i.e.*, include them multiple times in the plate of samples. Moreover, the genetic map presented here was constructed using only SNPs that mapped to the anchored portion of the reference genome to allow comparison between physical and genetic map positions. By including the unanchored portion of the genome sequence during the initial alignment stage, it is likely that additional SNPs could be identified and placed on the genetic map.

For 364 SNPs (18.3% of all mapped SNPs), the linkage group assignments conflicted with the predicted chromosomal locations according to the reference genome. Antanaviciute *et al.* (2012) reported a similar proportion (13.7%) using genotype data from the apple 8K SNP array. The most likely reasons for these conflicts between genetic and physical maps are the high frequency of paralagous genomic regions in the apple genome and the incorrect anchoring of sequences during the assembly of the reference genome. Such conflicts may obscure association signals and complicate the interpretation of genetic mapping results. For example, although below the conservative threshold for significance, we detected a signal of association on chromosome 13 when SNPs were positioned according to the physical map (Figure 3). However, this same block of SNPs that physically map to chromosome 13 was subsequently found to genetically map close to the QTL for skin color on linkage group 9 (Figure 4). This demonstrates that caution is warranted when relying on the physical map coordinates of the current reference genome sequence.

It is worth noting that our modest mapping population size of 87 F1 offspring likely often prevented the ordering algorithm from finding the correct order for SNPs that were closer than ∼2–5 cM. Over larger distances, however, the estimated map order of SNPs was generally in agreement with physical coordinates from the reference genome (Figure S2).

The present study demonstrates that a Mendelian trait (skin color) can be mapped in an apple F1 population using GBS data from a single lane of Illumina next-generation sequencing. Considering the currents costs of acquiring SNP data from the apple 8K genotyping microarray, we estimate that a similar quantity and quality of apple SNP data can be achieved using GBS with a 10–100× decrease in cost. Because of the rapid improvements in DNA sequencing technology, we anticipate that genotyping efforts will increasingly favor the use of GBS or similar methods over the use of microarrays. The present study uses standard software tools and simple heuristics to generate biological insights from GBS data. However, it is clear that methodological developments required for analyzing GBS data lag far behind the technology developed to generate GBS data. To maximize the utility of next-generation DNA sequence in the future, there is a clear need for improved computational and statistical tools to extract as much information as possible from the raw data, and to phase, impute, order, and genetically map large sets of genetic markers.

## Supplementary Material

Supporting Information
