# Supplementary material for: Fast and Cost-Effective Genetic Mapping in Apple Using Next-Generation Sequencing
Source: G3 (Bethesda). 2014 Jul 16;4(9):1681–7. doi: 10.1534/g3.114.011023 (PMC4169160; doi:10.1534/g3.114.011023)
Supplement: Supporting Information [file supp_4_9_1681__index.html]

Fast and Cost-Effective Genetic Mapping in Apple Using Next-Generation Sequencing — Supporting Information 

# Fast and Cost-Effective Genetic Mapping in Apple Using Next-Generation Sequencing

## Supporting Information for Gardner *et al.*, 2014

**Files in this Data Supplement:**

- Supporting Information - Figures S1-S3 (PDF, 287 KB)
- Figure S1 - A custom GBS analysis pipeline. (PDF, 147 KB)
- Figure S2 - A composite genetic linkage map of the *Malus x domestica* genome. (PDF, 209 KB)
- Figure S3 - Genome-wide LOD scores for apple fruit skin color. (PDF, 136 KB)
