# Supplementary material for: Fast and Cost-Effective Genetic Mapping in Apple Using Next-Generation Sequencing
Source: G3 (Bethesda). 2014 Jul 16;4(9):1681–7. doi: 10.1534/g3.114.011023 (PMC4169160; doi:10.1534/g3.114.011023)
Supplement: Supporting Information [file supp_g3.114.011023_011023SI.pdf]

G3 · Genes | Genomes | Genetics

Fast and cost-effective genetic mapping in apple using next-generation sequencing

Kyle M. Gardner*, Patrick Brown[§], Thomas F Cooke[†], Scott Cann*, Fabrizio Costa[‡], Carlos Bustamante[†], Riccardo Velasco[‡], Michela Troggio[‡] and Sean Myles*

* Department of Plant and Animal Sciences, Faculty of Agriculture, Dalhousie University, Nova Scotia, Canada
[§] Department of Crop Sciences, University of Illinois, Urbana, Illinois, USA
[†] Department of Genetics, Stanford School of Medicine, Stanford University, Stanford, California, USA
[‡] Genetics and Molecular Biology Department, IASMA Research Center, San Michele all'Adige, Italy

Data available via the Dryad Digital Repository: http://doi.org/10.5061/dryad.55t54

*Corresponding author:
Sean Myles
Department of Plant and Animal Sciences
Faculty of Agriculture
Dalhousie University
Kentville, Nova Scotia
B2N 5E3
Canada
Phone: +1-902-365-8460
Fax: +1-902-679-2311
Email: sean.myles@dal.ca

DOI: 10.1534/g3.114.011023

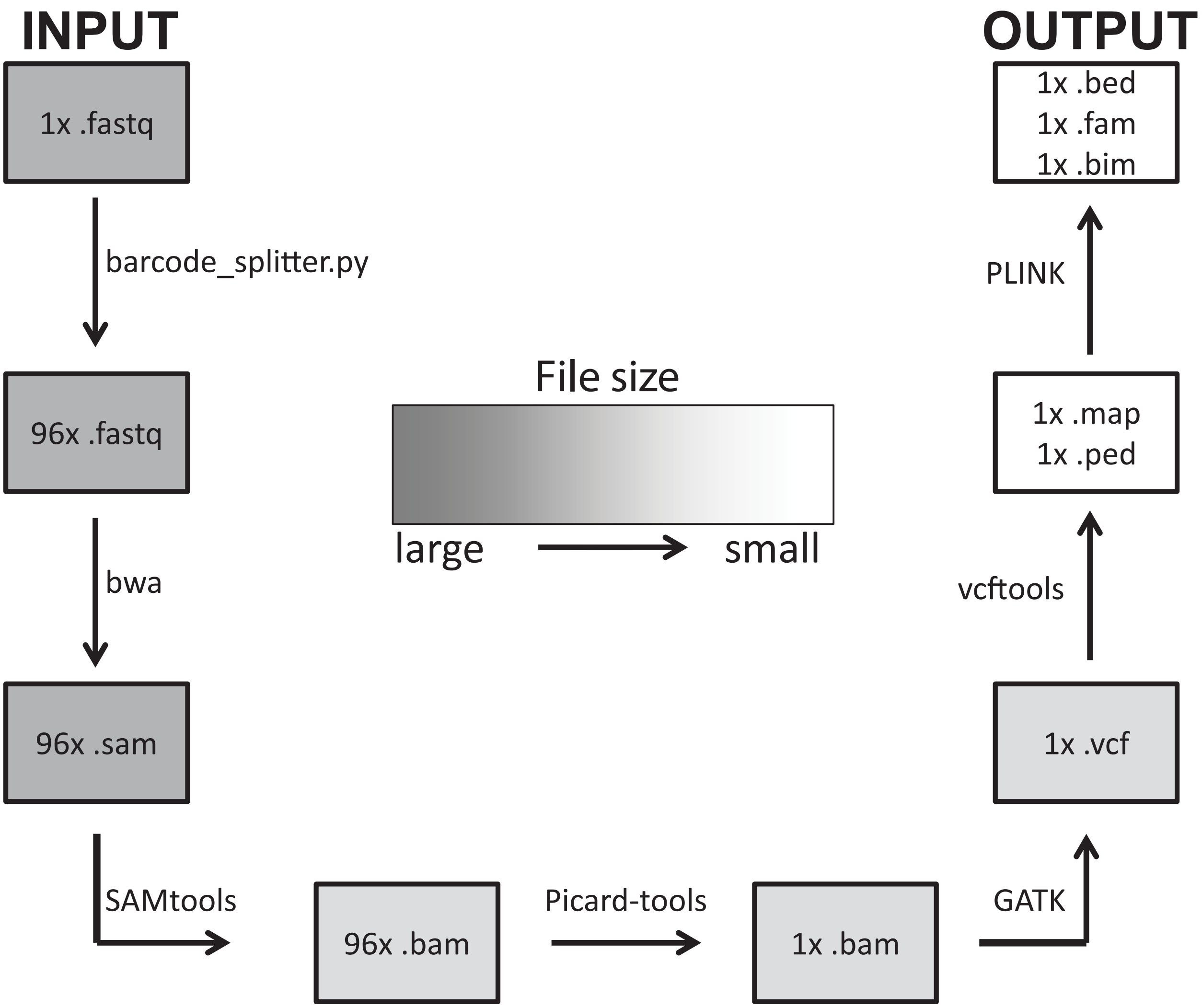

Figure S1 A custom GBS analysis pipeline. Each box represents a file type, indicated by its file extension, within the pipeline. Starting with a single .fastq file from the Illumina HiSeq 2000 sequencing machine and ending with a single set of PLINK files, the software packages and custom scripts used to move from one file type to another are indicated beside each arrow. Details of each step of the pipeline are provided in the Materials and Methods.

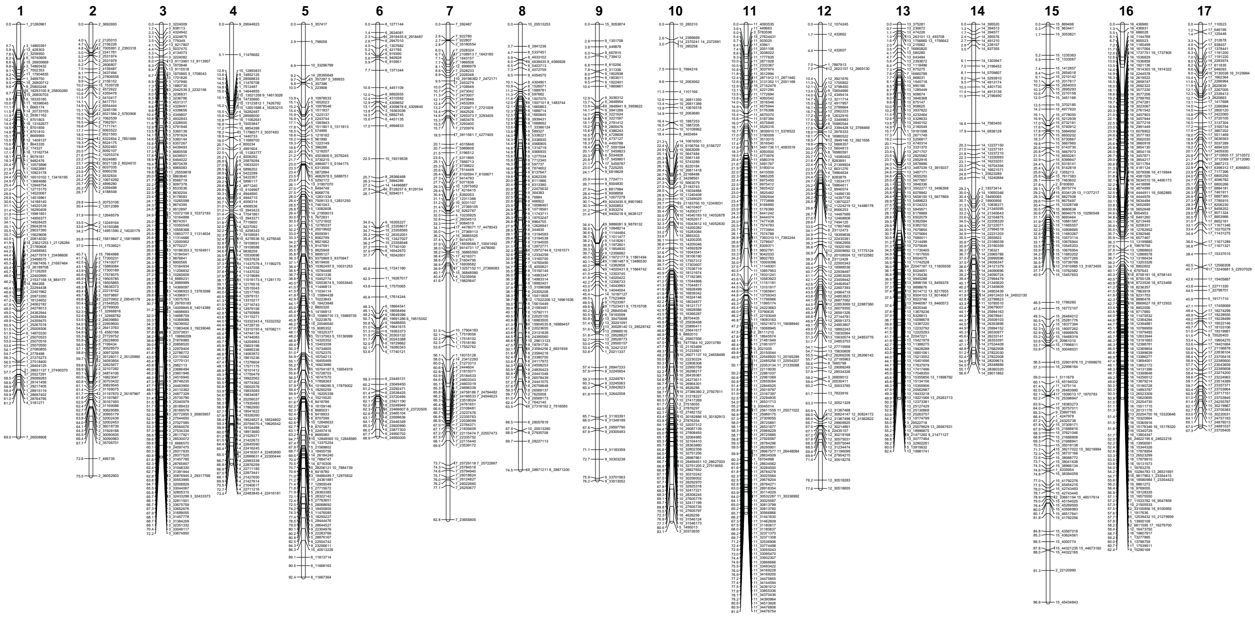

Figure S2 A composite genetic linkage map of the *Malus x domestica* genome. The map was constructed from 1994 SNPs discovered and genotyped using GBS, including SNPs from all three pseudo-testcross segregation types, in a Golden Delicious x Scarlet Spur F1 population.

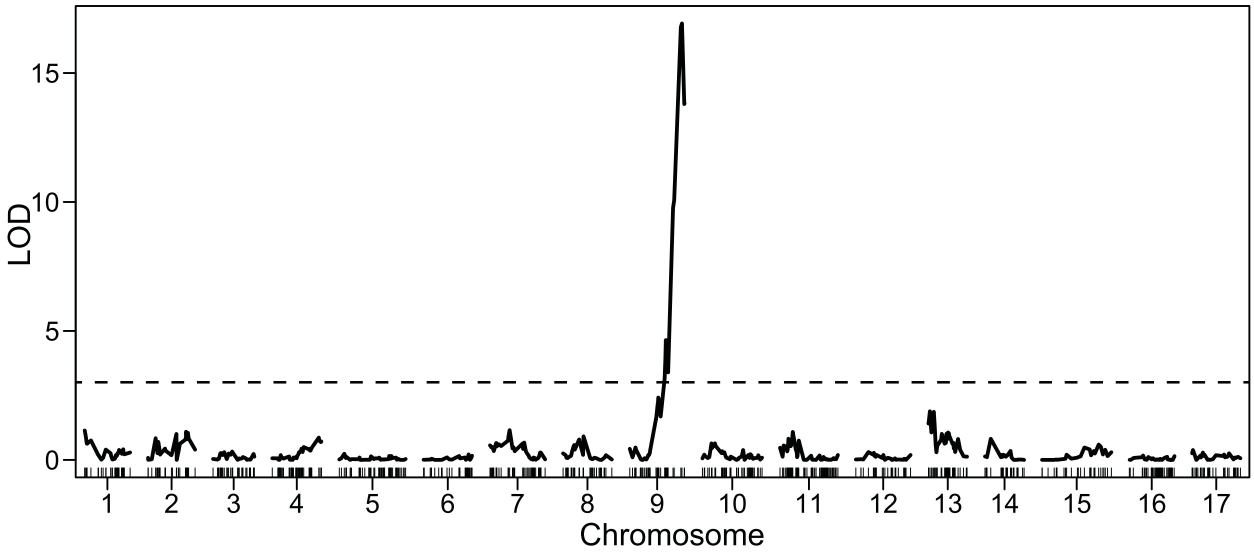

Figure S3 Genome-wide LOD scores for apple fruit skin color. Each SNP is represented by a single vertical line at the bottom of the plot. Only a single peak associated with skin color was identified on linkage group 9 and this peak overlaps with the known apple skin color locus. The horizontal dashed line represents the significance threshold determined by permutation.
