# Supplementary material for: Fast and Cost-Effective Genetic Mapping in Apple Using Next-Generation Sequencing
Source: G3 (Bethesda). 2014 Jul 16;4(9):1681–7. doi: 10.1534/g3.114.011023 (PMC4169160; doi:10.1534/g3.114.011023)
Supplement: Supporting Information [file supp_g3.114.011023_FigureS1.pdf]

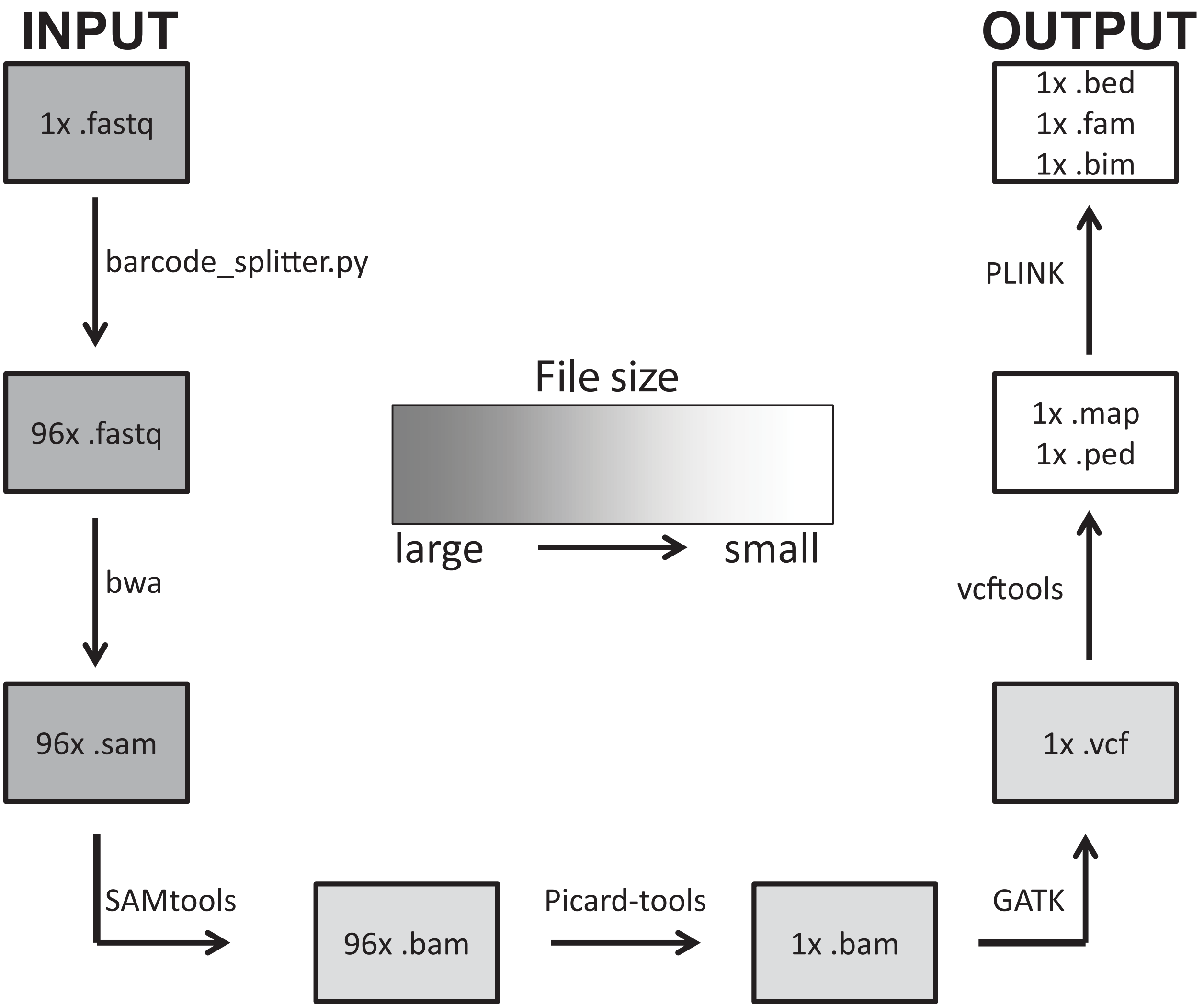

Figure S1 A custom GBS analysis pipeline. Each box represents a file type, indicated by its file extension, within the pipeline. Starting with a single .fastq file from the Illumina HiSeq 2000 sequencing machine and ending with a single set of PLINK files, the software packages and custom scripts used to move from one file type to another are indicated beside each arrow. Details of each step of the pipeline are provided in the Materials and Methods.
