# Supplementary material for: Fast and Cost-Effective Genetic Mapping in Apple Using Next-Generation Sequencing
Source: G3 (Bethesda). 2014 Jul 16;4(9):1681–7. doi: 10.1534/g3.114.011023 (PMC4169160; doi:10.1534/g3.114.011023)
Supplement: Supporting Information [file supp_g3.114.011023_FigureS2.pdf]

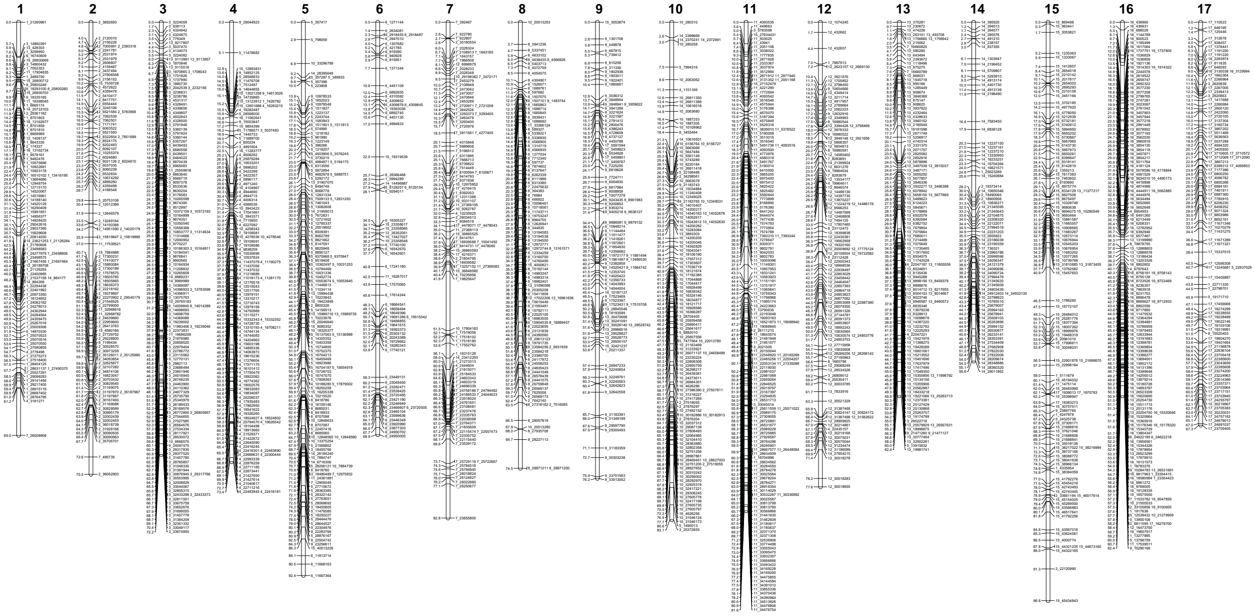

Figure S2 A composite genetic linkage map of the *Malus x domestica* genome. The map was constructed from 1994 SNPs discovered and genotyped using GBS, including SNPs from all three pseudo-testcross segregation types, in a Golden Delicious x Scarlet Spur F1 population.
