# Supplementary material for: Fast and Cost-Effective Genetic Mapping in Apple Using Next-Generation Sequencing
Source: G3 (Bethesda). 2014 Jul 16;4(9):1681–7. doi: 10.1534/g3.114.011023 (PMC4169160; doi:10.1534/g3.114.011023)
Supplement: Supporting Information [file supp_g3.114.011023_FigureS3.pdf]

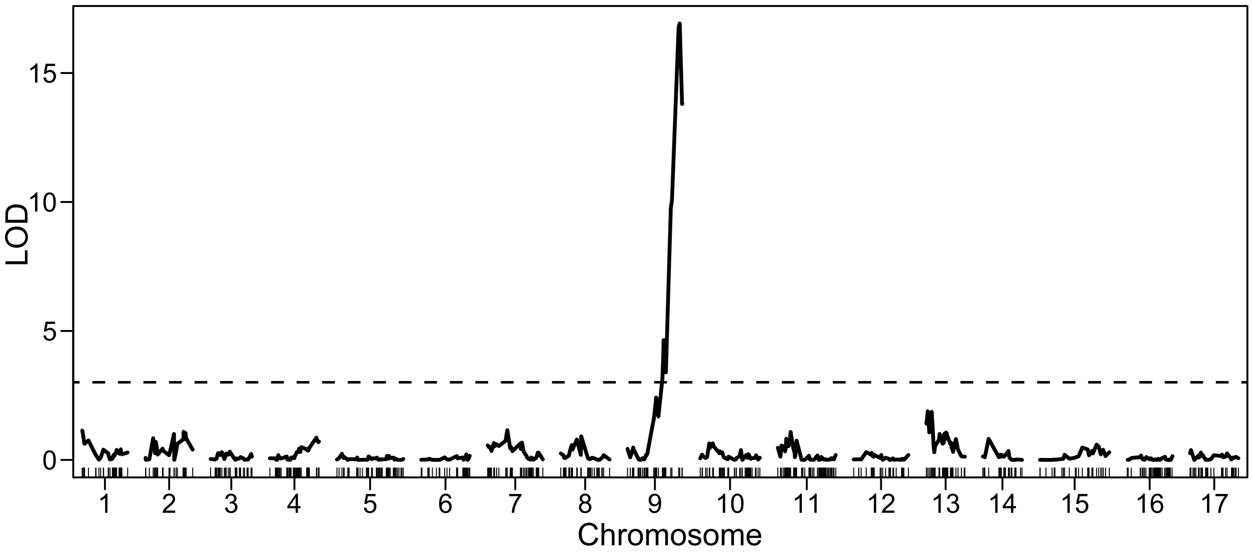

Figure S3 Genome-wide LOD scores for apple fruit skin color. Each SNP is represented by a single vertical line at the bottom of the plot. Only a single peak associated with skin color was identified on linkage group 9 and this peak overlaps with the known apple skin color locus. The horizontal dashed line represents the significance threshold determined by permutation.
